# RNA Sequencing Reveals that Endoplasmic Reticulum Stress and Disruption of Membrane Integrity Underlie Dimethyl Trisulfide Toxicity against *Fusarium oxysporum* f. sp. *cubense* Tropical Race 4

**DOI:** 10.3389/fmicb.2017.01365

**Published:** 2017-07-24

**Authors:** Cunwu Zuo, Weina Zhang, Zhongjian Chen, Baihong Chen, Yonghong Huang

**Affiliations:** ^1^College of Horticulture, Gansu Agricultural University Lanzhou, China; ^2^Institute of Fruit Tree Research, Guangdong Academy of Agricultural Sciences Guangzhou, China; ^3^Agro-biological Gene Research Center, Guangdong Academy of Agricultural Sciences Guangzhou, China; ^4^College of Horticulture, Qingdao Agricultural University Qingdao, China

**Keywords:** Dimethyl trisulfide (DT), *Fusarium oxysporum* f. sp. *cubense* tropical race 4 (*Foc* TR4), target sites, endoplasmic reticulum (ER) stress, steroid biosynthesis

## Abstract

Fusarium wilt of banana, a destructive disease that affects banana production, is caused by *Fusarium oxysporum* f. sp. *cubense* tropical race 4 (*Foc* TR4). In a previous study, we confirmed the strong inhibitory effects of Chinese leek (*Allium tuberosum*) on the incidence of this disease. Sulfur compounds are the primary antifungal constituents of Chinese leek. Among these, dimethyl trisulfide (DT) was the most abundant and exhibited the strongest inhibition of *Foc* TR4 growth and development. In the present study, the global gene expression profiles of *Foc* TR4 isolates treated with DT at 4,000-folds dilution (concentration of 1/4,000, v/v) for 1.5, 6, and 12 h were investigated by using RNA sequencing. The expression patterns of 15 DEGs were validated based on quantitative real-time PCR (qRT-PCR) assay. Untreated sample presented 2,556, 1,691, and 1,150 differentially expressed genes (DEGs) at 1.5, 6, and 12 h after the onset of the experiment, respectively, whereas DT-treated isolates presented 2,823, 3,546, and 6,197 DEGs. Based on Gene Ontology (GO) annotation and Kyoto Encyclopedia of Genes and Genomes (KEGG) enrichment analysis, DEGs involved in endoplasmic reticulum (ER), glycosylation, and steroid biosynthesis were significantly inhibited by DT exposure. The similar expressional patterns of 15 DEGs between RNA-seq and qRT-PCR assays indicated the reliability of the RNA-seq data. In conclusion, ER stress related to glycosylation inhibition and damage to cell membrane integrity might contribute to the toxicity of DT against *Foc* TR4. As the results presented here evidenced changes in gene expression associated with DT exposure, which might be used to develop new approaches for controlling FWB.

## Introduction

*Fusarium oxysporum*S has been considered one of the most prevalent fungal pathogens, having a wide host range and causing severe losses in multiple crops such as tomato, cotton, maize, and banana (Dean et al., 2012; Aiyaz et al., 2016; Nirmaladevi et al., 2016). FWB, caused by *Foc*, is one of the most destructive diseases affecting banana cultivation worldwide (Ploetz, 2015), and has led to considerable losses in production since it was first discovered in Austria in 1876 (Ploetz and Pegg, 1997, 2000). It is currently found in all banana-producing regions of the world, including southern Asia, Africa, and Latin America (García-Bastidas et al., 2014; Ordonez et al., 2015; Ploetz, 2015). The pathogen has divided into 4 races and 24 VCGs, based on host type and vegetative compatibility, respectively (Das et al., 2012; Fourie et al., 2009). Among these, *Foc* tropical race 4 (*Foc* TR4, VCG 01213/16) is one of the most concern owing to its wide host range and strong pathogenicity (Li et al., 2012).

Resistance breeding is traditionally regarded as the most durable, environmentally friendly, and convenient control practice (Hwang and Ko, 2004). However, owing to the long cultivation cycle of banana and the rapid evolution of *Foc*, few resistant cultivars have been used in the field to date (Ploetz, 2015). Considerable interest is therefore focused on the exploitation of naturally occurring organisms, such as nonpathogenic *F. oxysporum, Burkholderia cenocepacia, Bacillus amyloliquefaciens*, and *Streptomyces albospinus* for the control of crop diseases including FWB (Postma and Rattink, 1992; Raguchander et al., 1997; Butt et al., 2001; Fravel et al., 2003; Cao et al., 2005; Asha et al., 2011a,b; Wang et al., 2013; Ho et al., 2015). Furthermore, many antifungal secondary metabolites have been identified from plants and microorganism (Paiva et al., 2010; Coleman et al., 2011), and the identification of novel antifungal targets for use as control agents is currently becoming an important strategy (De Backer and Van Dijck, 2003; Walsh et al., 2010). Some of these targets include chitin, the major component of the fungal cell wall, and ergosterol, which is essential to membrane formation. These components, being absent in most mammalian and plant cells, have been considered as main targets of antifungal compounds to prevent and control fungal infections (Behr, 2011; Alcazar-Fuoli and Mellado, 2013). However, the long-term intensive use of single target inhibitors often results in the enhancement of fungal drug resistance. Therefore, it is urgent to identify alternative therapeutics for future use.

It is also crucial to investigate the mechanisms by which these compounds exert their fungicidal activity, not only for discovery of new antifungal substances and identification of their target sites, but also for risk assessment (Ma and Michailides, 2005). The emergence of high-throughput sequencing technologies and expansion of genomic information has provided new methodologies for the investigation of antifungal mechanisms and identification of potential targets (Cools and Hammond-Kosack, 2013). Numerous studies regarding the response of fungal gene expression profiles to plant essential oils have been conducted, and potential targets such as cell wall-, cell membrane- and secondary metabolism-related genes were found (Parveen et al., 2004; Yu et al., 2010). These results have supplied information that contributes to understanding the antifungal mechanisms of plant essential oils. However, systematic studies on the mechanism of toxicity of such compounds to *Foc* have been limited.

Recently, we demonstrated the significant inhibitory effect of the Chinese leek (*Allium tuberosum*) on the occurrence of FWB in the field (Huang et al., 2012). Furthermore, the strong inhibitory effects of Chinese leek root exudates and tissue extracts on *Foc* growth have also been confirmed using *in vitro* tests (Huang et al., 2012; Zuo et al., 2015), and the strong inhibitory effects of Chinese leek extracts and secondary metabolites on other pathogenic microorganisms and nematodes have been verified (Lee et al., 2004; Tsao and Yin, 2001; Irkin and Korukluoglu, 2007; Huang et al., 2016). Studies on the mechanism of toxicity of the secondary metabolites of Chinese leek revealed that they caused ROS burst and decrease of mitochondrial membrane potentials in *Foc*TR4 cells (Zuo et al., 2015). The expression of the ergosterol biosynthesis genes CYP51-2 and CYP51-3 was reduced but that of the autophagy related genes ATG 1, ATG 8, and ATG 15 was induced at the early stage of the treatment of *Foc* cells with Chinese leek root exudates (Zuo et al., 2015). Sulfur and phenolic compounds were determined to be the primary antifungal compounds in Chinese leek; of these, DT was the principal component among the sulfur compounds and showed strong inhibitory effects on *Foc* growth and development (Zhang et al., 2013; Zuo et al., 2015).

In the present study, we firstly confirmed the toxicity of DT to *Foc* TR4. Further, to explore the molecular mechanism(s) underlying this toxicity and to identify the major target sites involved, global gene expression profiles of *Foc* TR4 at three time points with or without DT treatment were investigated using RNA-seq.

## Materials and Methods

### Fungal Isolates and Chemicals

Isolates of the pathogenic fungus *Foc* tropical race 4 (*Foc* TR4, ACCC 37982), which causes FWB, was obtained from diseased Cavendish ‘Brazilian’ (AAA) plants with FWB symptoms in Guangdong Province, China. Standard DT was purchased from Sigma-Aldrich Co. LLC (St. Louis, MO, United States).

### Toxicity Test

The *Foc* TR4 isolates were cultured on potato dextrose agar for 5 days at 28°C in the dark. Conidia were then collected and transferred into PDB, and the final concentration of these suspensions was adjusted to 4 × 10^6^ conidia/ml. For the toxicity test, DT were added into conidial suspensions, which were diluted 2,000, 4,000, 8,000, and 16,000 folds, with the final concentration were 1/2,000, 1/4,000, 1/8,000, and 1/16,000, respectively. Then the treated conidia were cultured for 6, 12, 24, or 48 h. The viability of conidia was analyzed using the FDA assay (Tolosa et al., 2015). In brief, control and treated conidia were incubated with 50 μM FDA (Life Technologies Inc., Gaithersburg, MD, United States) in the dark, for 5 min at 28°C. To remove redundant dye, conidia were washed three times in PBS (pH7.0). Qualitative analysis was performed using a multifunctional microplate reader (Varioskan Flash;Thermo Scientific, Wilmington, DE, United States) with an excitation wavelength of 488 nm and an emission wavelength of 530 nm.

### RNA Isolation and RNA-seq Library Preparation and Sequencing

To further understand the molecular mechanism of the potential antifungal action, the global gene expression profiles at 1.5, 6, and 12 h of conidia treated with DT at 1/4000 (treated group; 1.5-T, 6-T, and 12-T) and untreated conidia (control group; 1.5-C, 6-C, and 12-C) were investigated by using RNA-seq. Conidia in the control group at 0 h (0-C) were used as the control. All conidia were collected by centrifugation, and total RNA was isolated by using the Fungi RNAout kit (60305-50, TiandzInc., Beijing, China). The quality and quantity of RNA was determined using 1.5% denatured agarose gel electrophoresis and a NanoDrop 2000 Spectrophotometer (Thermo Scientific), and an Agilent 2100 Bioanalyzer (Agilent Technologies, Palo Alto, CA, United States), respectively. Poly(A) mRNA was isolated by using oligo (dT) magnetic beads and then fragmented into short segments. First-strand cDNA was then synthesized by using a random hexamer-primer, whereas second strand cDNA was synthesized by using DNA polymerase I and RNase H. Double-strand cDNA was purified by using magnetic beads. End-repair and 3’-end single nucleotide A (adenine) addition were performed using T4 DNA polymerase and Klenow 3’- to 5’- exo-polymerase, respectively. Sequencing adaptors were ligated to the fragments using T4 quick ligase; the fragments were then enriched by using PCR amplification. The quality of the sample library and its quantification were obtained using the Agilent 2100 Bioanaylzer and an ABI StepOnePlus Real-Time PCR System (ABI, CA, United States). The cDNA library were sequenced via Illumina HiSeq^TM^ 2000.

### Assembly Screening for DEGs, GO Annotation, and KEGG Enrichment

We use *SOAPnuke v1.5.2* and ‘-l 5 -q 0.3 -n 0.1’ for quality control and reads filtering, respectively. After that, clean reads were mapped to the genome sequence of *Foc II5* (Fusarium Comparative Sequencing Project, Broad Institute of MIT and Harvard^1^). Gene expression levels were quantified based on the RSEM *v1.2.20* with bam file alignment by *Bowtie2 v2.2.3* and default *RSEM* parameters (RNASeq by Expectation Maximization) (Li and Dewey, 2011). Gene identification (gene ID), length, log2 ratio, false discovery rate (FDR), GO classification, and KEGG orthology were obtained. The DEGs were screened based on the Poisson distribution method (Audic and Claverie, 1997). We used a FDR ≤ 0.01 and an absolute value of log2 ratio ≥ 2 as thresholds judge the significance of differences in gene expression. The GO classification of DEGs and the distribution analysis of gene function in species at the macro level were conducted using WEGO software (Ye et al., 2006), whereas pathway enrichment analysis was obtained from KEGG (Kanehisa et al., 2008). GO categories with a level equal to “5” and “FDR < 0.05” in each sample were screened while six most enriched pathways were selected for further investigation.

### Quantitative Real-Time PCR

Fifteen DEGs were further confirmed by using qRT-PCR. In brief, the total RNA from each sample was purified and converted into cDNA using the PrimeScript^TM^ RT reagent Kit with gDNA Eraser (Code No. RR047A, TAKARA Biotechnology CO., LTD., Dalian, China). Primer pairs were designed for each DEG by using the online software program Primer 3^2^ (primer sequences are listed in **Supplementary Table S1**). The qRT-PCR was conducted on a CFX96 Touch^TM^ Real-Time PCR Detection System (Bio-Rad, United States) by using SYBR^®^Premix ExTaq^TM^ II (Tli Rnase H Plus; TAKARA Biotechnology CO., LTD.). The specificity of primers was assessed by using the dissociation curve method. Relative gene expression was normalized to that of actin (López-Berges et al., 2010) and calculated by the comparative –ΔΔCt method. Data from toxicity tests and qRT-PCR analysis were expressed as means ± SD of three independent replicates.

## Results

### Inhibition of *Foc* TR4 Growth by DT

**FIGURE 1:**
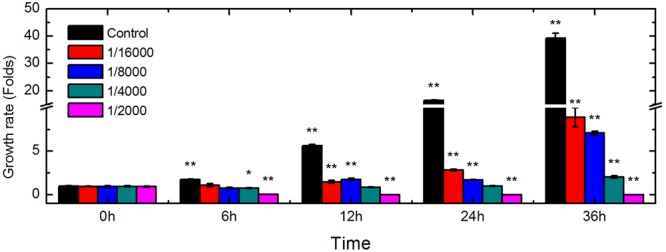
Changes in *Foc* TR4 viability induced by DT exposure. Conidia from control and treated isolates were incubated with FDA dye and their viability was assessed using a multifunctional microplate reader. Asterisks indicate a significant difference from the control, at ^∗^*P* < 0.05, and ^∗∗^*P* < 0.01.

To determine the inhibitory effects of DT on the growth of *Foc* TR4, the proliferation of conidia in control and treated groups was tested at various time points (**Figure 1**). In the untreated group (without DT), conidial viability increased to 1.78-, 5.67-, 16.44-, and 39.26-fold that of the control (0 h), after being cultured for 6, 12, 24, and 36 h, respectively. The growth ratio was significantly inhibited by DT at concentrations up to 1/16,000 (v/v). At this concentration, viability increased to 1.12-, 1.49-, 2.85-, and 8.9-fold compared to the control at 6, 12, 24, and 36 h of culture, respectively, which were much lower than that of the untreated group at the same time point. Inhibitory effect was correlated with the concentrations of DT. The growth of the *Foc* TR4 were almost entirely inhibited by 1/4,000 DT treated for 24 h and viability was 0.78-, 0.86-, and 1.02-fold that of the untreated group at 6, 12, and 24 h, respectively. In addition, with 1/2,000 DT, the viability of *Foc* TR4 conidia were decreased to 4% that of the untreated conidia at 6 h, and no conidia survived after 12 h incubation at this concentration.

### Transcriptomic Profiles of *Foc* TR4 Influenced by DT

**FIGURE 2:**
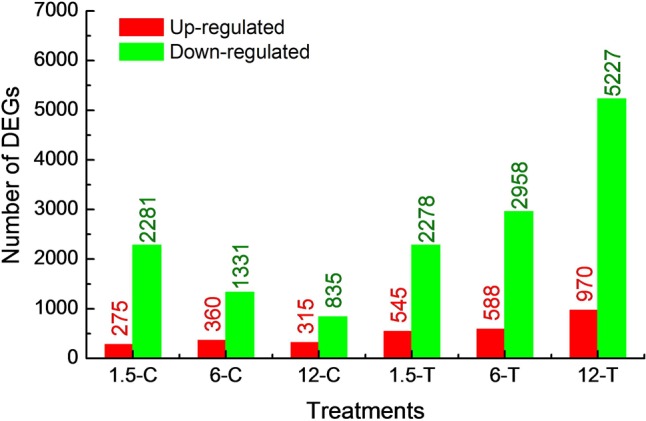
Number of DEGs, which were up- and down-regulated in control and treated groups.

The global gene expression profile of *Foc* TR4 treated or untreated with DT for three different periods (1.5, 6, and 12 h) were examined using RNA-seq. The raw reads have been deposited at the Sequence Read Archive (SRA) dababase of NCBI (SRR5724804 to SRR5724810). After filtering, about 10 million clean reads were obtained from each sample, of which nearly 90% were mapped to the reference genome (**Table 1**). Gene mapping rate and number of expressed genes ranged from 43.03 to 73.12% and from 11,137 to 13,305, respectively (**Table 1**). At 1.5, 6, and 12 h, 2,556, 1,691, and 1,150 DEGs were found in the control group and 2,823, 3,546, and 6,197 DEGs were found in the treated group (FDR ≤ 0.01; log2 ratio ≥ 2 or ≤ -2). Most DEGs were down-regulated in both control and treated groups. The DEGs identified within each sample was used for functional classification and annotation (**Figure 2**). The detail of all DEGs were supplied at **Supplementary Table S2**.

**Table 1:** Summary data of reads in untreated and treated group.

Sample name	Clean reads	Genome map rate (%)	Gene map rate (%)	Expressed gene
0-C	10, 114, 056	92.08	67.98	13, 305
1.5-C	10, 220, 073	88.31	71.25	12, 079
6-C	10, 410, 250	90.57	73.12	12, 646
12-C	10, 165, 012	90.66	71.39	12, 930
1.5-T	10, 290, 574	91.58	71.80	12, 310
6-T	10, 279, 749	92.24	67.73	11, 137
12-T	10, 144, 814	92.71	43.03	11, 417

*For sample name, 0-C is Control; 1.5-C, 6-C and 12-C presents *Foc* TR4 cultured in PDB medium for 1.5, 6, and 12 h, respectively; 1.5-T, 6-T, and 12-T indicates *Foc* TR4 treated by DT for 1.5, 6, and 12 h, respectively.*

### GO Analysis: Functional Categorization of Identified DEGs

**FIGURE 3:**
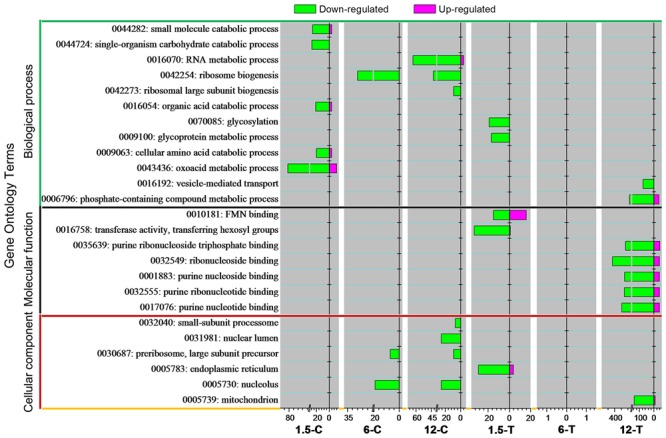
Gene Ontology classification analysis of DEGs. GO terms for which the false discovery rate (FDR) value was lower than 0.05 are listed.

In total, 12, 6, and 7 GO terms were distributed into BP, MF, and CC, respectively (**Figure 3**). A large number DEGs (3,546) were found from 6-T, indicated the multiple responses was induced. However, enriched GO term was yet to be discovered. To understand the molecular mechanism underlying DT toxicity, the early responses of *Foc* TR4 to this compound were first investigated. At an early stage (1.5 h), the categories “small molecule catabolic process (GO: 0044282)”, “single-organism carbohydrate process (GO: 0044724)”, “organic acid catabolic process (GO: 0016054)”, “cellular amino acid catabolic process (GO: 0009063)”, and “oxacid metabolic process (GO: 0043436)” belonging to BP were clearly enriched in the control group (1.5-C). However, the categories “glycosylation (GO: 0070085)” and “glycoprotein metabolic process (GO: 0009100)” belonging to BP, “FMN binding (GO: 0010181)” and “transferase activity, transferring bexosyl groups (GO: 0016758)” belonging to MF, and “ER (GO: 0005783)” belonging to CC were considerably affected in the treated group (1.5-T). At later stages, the categories “ribosome biogenesis (GO: 0042254)” within BP, and “preribosome, large-subunit precursor (GO: 0030687)” and “nucleolus (GO: 0005730)” within CC were enriched in the control group (6-C and 12-C). In contrast, multiple categories such as “vesicle-mediated transport (GO: 0016192)” and “mitochondrion (GO: 0005739)” within CC were significantly enriched 12 h after DT treatment.

### Pathway Enrichment Analysis of DEGs

**FIGURE 4:**
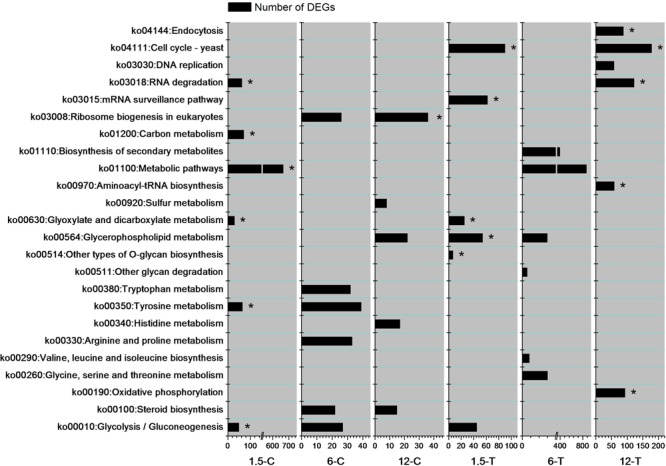
The six most enriched DEGs pathways in each sample. Asterisks indicate the pathways for which the FDR value was lower than 0.05.

To investigate the biological pathways involved in the mechanism of DT toxicity to *Foc* TR4, the six most enriched DEGs pathways from each sample were examined (**Figure 4**). At 1.5 h, the DEGs involved in “RNA degradation (ko03018)”, “carbon metabolism (ko01200)”, “metabolic pathways (ko01100)”, and “tyrosine metabolism (ko00350)” were specifically enriched in the control group, while “cell cycle-yeast (ko04111)”, “mRNA surveillance pathway (ko03015)”, “glycerophospholipid metabolism (ko02564)”, and “other types of *O*-glycan biosynthesis (ko00514)” were specifically increased in the DT-treated group. At 12 h, multiple pathways were modified in both the control and treated groups. However, “ribosome biogenesis in eukaryotes (ko03008)” was significantly enriched in the control group, while pathways such as “endocytosis (ko04144)”, “cell cycle-yeast (ko04111)”, “RNA degradation (ko03018)”, “aminocyl-tRNA biosynthesis (ko00970)”, and “oxidative phosphorylation (ko00190)” were enriched in the DT-treated group. Moreover, “ribosome biogenesis in eukaryotes”, “tyrosine metabolism”, and “steroid biosynthesis” were specifically enriched at two time points in the control group. Cell cycle-yeast (ko04111) was discovered from top six enriched pathways in 1.5-T and 12-T but not in 6-T. However, a large number of DEGs (97) involved in this pathway were detected from 6-T (data not shown). That indicate genes involved in cell cycle-yeast of *Foc* TR4 were strongly influenced by DT exposure.

### Down-Regulation of DEGs Related to ER Following DT Exposure

**FIGURE 5:**
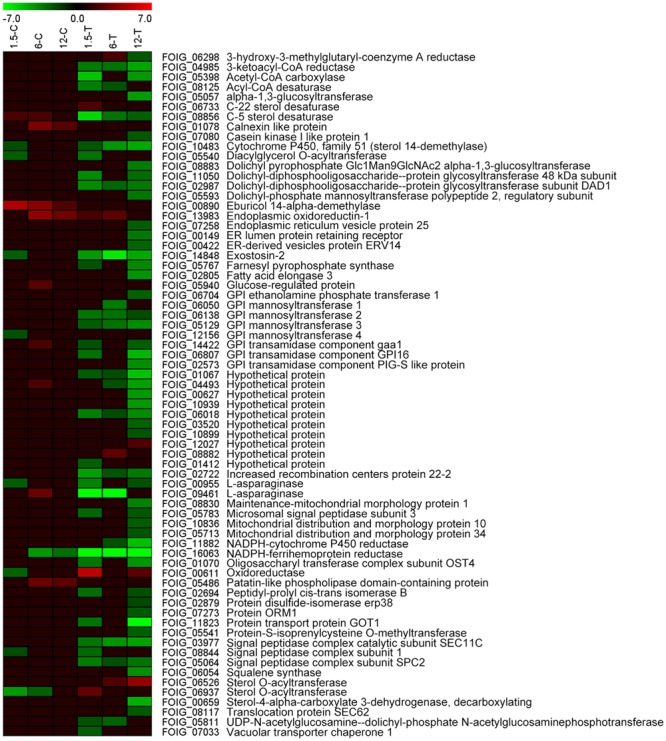
Expression profiles of ER-related genes.

A comprehensive illustration of DEGs related to the ER in *Foc* TR4, which were influenced by DT exposure, is presented in **Figure 5**. In total, 69 genes were differentially expressed in at least one of the samples. In the control groups, 2 out of 10, 9 out of 11, and 4 out of 5 genes were up-regulated in 1.5-C, 6-C, and 12-C, respectively, whereas 34, 23, and 49 DEGs were identified in1.5-T, 6-T, and 12-T treated groups, respectively, most of them down-regulated. Notably, a large number of DEGs found in treated groups were obviously down-regulated at the early stage of DT exposure. These included 3-ketoacyl-CoA reductase (FOIG_04985), Acetyl-CoA carboxylase (FOIG_05398), C-5 sterol desaturase (FOIG_08856), protein glycosyltransferase (FOIG_11050 and FOIG_02987), farnesyl pyrophosphate synthase (FOIG_05767), GPI mannosyltransferase (FOIG_06138 and FOIG_05129), increased recombination centers protein 22-2 (FOIG_02722), L-asparaginase (FOIG_09461), microsomal signal peptidase subunit 3 (FOIG_05783), NADPH-ferrihemoprotein reductase (FOIG_16063), oligosaccharyl transferase complex subunit OST4 (FOIG_01070), signal peptidase complex (FOIG_03977 and FOIG_05064), phosphotransferase (FOIG_05811), and vacuolar transporter chaperone 1 (FOIG_07033) (**Figure 5**).

### Repression of DEGs Involved in Steroid Biosynthesis Following DT Exposure

**FIGURE 6:**
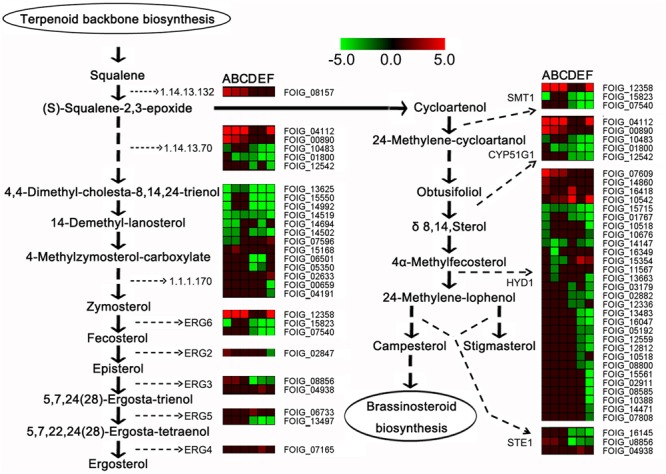
Expression profiles of steroid biosynthesis-related genes. In the heatmap, (A–C) represent 1.5-C, 6-C, and 12-C (controls after 1.5, 6, and 12 h of incubation), and (D–F) represent 1.5-T, 6-T, and 12-T (treated samples after 1.5, 6, and 12 h of DT exposure), respectively.

The expression of the genes involved in steroid biosynthesis was considerably repressed following DT exposure (**Figure 6**). These genes encoded several enzymes, including EC 1.14.13.132 (FOIG_08157), CYP51G1 or EC 1.14.13.70 (FOIG_04112, FOIG_00890, FOIG_01800 and FOIG_12542), and EC 1.1.1.170 (FOIG_06501 and FOIG_02633). Moreover, the expression of genes encoding enzymes involved in ergosterol biosynthesis, such as ERG 6 (FOIG_12358, FOIG_07540), ERG 2 (FOIG_02847), ERG 3 (FOIG_8856), and ERG 5 (FOIG_13497), was clearly reduced following DT exposure.

### Verification of DEGs Using qRT-PCR

**FIGURE 7:**
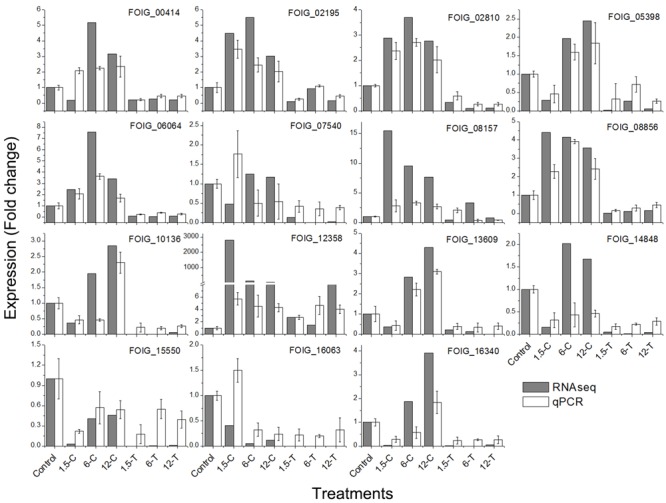
Quantitative real-time PCR validation of candidate genes in enriched GO terms and pathways. Genes involved in: endocytosis, FOIG_02195, FOIG_00414, FOIG_13609, and FOIG_02810; oxidative phosphorylation, FOIG_06064; steroid biosynthesis, FOIG_07540, FOIG_08157, FOIG_12358, andFOIG_15550; and ER, FOIG_05398, FOIG_08856, FOIG_14848, and FOIG_16063.

To confirm the accuracy of the RNA-seq results, the expression patterns of 15 representative DEGs involved in enriched GO terms or pathways were verified by qRT-PCR assay (**Figure 7**). These included DEGs involved in steroid biosynthesis (FOIG_07540, FOIG_08157, FOIG_12358, and FOIG_15550), and related to the ER (FOIG_05398, FOIG_08856, FOIG_14848, and FOIG_16063). The changes observed in trends were similar between RNA-seq and qRT-PCR assays, although several genes presented different expression at the same time point. This indicated the reliability of the RNA-seq data.

## Discussion

The aim of this study was to examine the toxicity of DT against *Foc* TR4 and the molecular mechanisms underlying it; our results confirmed that the antifungal activity of DT against this pathogen and showed the dynamic gene expression profiles of the *Foc* TR4 response to DT. The GO and KEGG analysis indicated that the DEGs involved in ER and steroid biosynthesis were significantly enriched following DT exposure. At a later stage of DT exposure, DEGs associated with preribosome, endocytosis, RNA degradation, and oxidative phosphorylation were also notably influenced by DT. To the best of our knowledge, this is the first investigation of global gene expressions of *Foc* TR4 in response to DT exposure.

### ER Stress Was Induced in *Foc* TR4 at an Early Stage of DT Exposure

The ER is not only responsible for the synthesis and folding of most secretory and transmembrane proteins, but also perceives and responds to cellular biotic and abiotic stresses, i.e., to ER stress (Zhang and Kaufman, 2008). In multiple fungal and mammalian cells, ER stress is induced by several compounds, such as cisplatin and cadmium, and could be considered as a target for the toxicity of those compounds (Mandic et al., 2003; Gardarin et al., 2010). In the present study, the DEGs involved in ER were significantly enriched from 1.5-T onward, and most of these were down-regulated (**Figures 3, 5**). The expression of four genes, namely FOIG_05398, FOIG_08856, FOIG_14848, and FOIG_16063, was also validated by qRT-PCR (**Figure 7**), suggesting that ER stress is induced in *Foc* TR4 at an early stage of DT exposure.

In general, ER stress is characterized by an accumulation of unfolded proteins in the ER, which results in the unfold protein response (UPR) (Zhang and Kaufman, 2008). This can be induced by the inhibition of glycosylation, rapid generation of ROS and perturbation of calcium metabolism (Yokouchi et al., 2008; Gardarin et al., 2010). Glycosylation, a post-translational modification of proteins on the ER or ribosome, plays important roles in many cellular events, both structural and signal recognition-related (Hart, 1992). In fungi, glycosylation is involved in the synthesis of the cell wall, cryptococcal capsule, glycoproteins, and glycolipids (Klutts et al., 2006). The expression of *Foc* TR4 genes involved in glycosylation and in glycoproteins metabolic processes was significantly reduced following DT treatment (**Figure 3**, and **Supplementary Table S3**). A rapid ROS burst was also reported from *Foc* TR4 treated with Chinese leek root exudates (Zuo et al., 2015). However, a reduction in the expression of genes related to calcium influx, such as calnexin like protein (FOIG_01078), were enriched in at the later stage of DT exposure (**Figure 5**). Therefore, ER stress in *Foc* TR4 resulting from DT exposure might be related to glycosylation inhibition and ROS accumulation, but it is likely not involve the perturbation of calcium influx homeostasis.

Under normal conditions, ER stress triggers vital responses for cell survival, including autophagy, RNA degradation, and apoptosis (Bonilla et al., 2002; Tabas and Ron, 2011; Hetz, 2012). Cell death under ER stress depends on the core mitochondrial apoptosis pathway (Hetz, 2012). At the later stage of DT exposure, DEGs involved in endocytosis, RNA degradation, and oxidative phosphorylation were enriched (**Figure 4**). As evidenced by the expression profiles of these DEGs, most of them were down-regulated in 12-T but not differentially expressed in other samples (**Supplementary Table S3**). This is consistent with the reduction in the mitochondrial transmembrane potential of *Foc* TR4 induced by Chinese leek root exudates (Zuo et al., 2015). Therefore, mitochondrial damage in *Foc* TR4, probably resulting from ER stress, was induced at a later stage of DT exposure. These results indicate that ER stress of *Foc* TR4 results in the activation of multiple stress responses, such as autophagy, RNA degradation, and mitochondrial damage.

### DT Might Affect *Foc* TR4 Cell Membrane Integrity

In addition to ER, several target sites, such as the cell wall, fungal sterols, and nucleic acids, have been proposed to account for the activity of antifungal compounds (Ghannoum and Rice, 1999). Plant essential oils or their volatile compositions have strong antifungal activity against *Penicillium digitatum, P. italicum*, and *Galactomyces citriaurantii* (Droby et al., 2008; Wuryatmo et al., 2003, 2014). The cell membrane, mitochondrion, and genetic material have also been considered major targets of volatile compound toxicity (Bakkali et al., 2008; Shao et al., 2013). Sterol and glycerophospholipids are important cellular or subcellular membrane components and are required for cell growth and proliferation, maintenance of organelle morphology, signal transduction, and lipid homeostasis (Kihara and Igarashi, 2004; Nebauer et al., 2004). Ergosterol is one of the principal sterol components in the fungal cell membrane; blocking ergosterol biosynthesis usually leads to the disruption of cell structure and function, and even to cell death (OuYang et al., 2016). Our results demonstrated that the DEGs involved in glycerophospholipids metabolism and steroid biosynthesis were highly enriched in 6-C and 12-C (**Figures 4, 6**). Among these, the relative expression of FOIG_07540, FOIG_08157, FOIG_12358, and FOIG_15550 was verified by qRT-PCR (**Figure 7**). Notably, the genes encoding several key enzymes responsible for ergosterol biosynthesis, including ERG3, ERG5, ERG2, ERG6, and CYP51G1 (ERG11) exhibited much lower expression levels in the treated group than in the control group (**Figure 7**). A previous study confirmed that the ergosterol biosynthesis pathway is essential for *Foc* TR4 conidial germination and that it could be considered an important target for FWB control (Deng et al., 2015). Reduced expression of the genes involved in ergosterol biosynthesis usually results in a decrease in ergosterol content in cells, causing osmotic disturbances and the disruption of cell growth and proliferation (Khan et al., 2010; Sun et al., 2011). Regarding the considerable influence of DT exposure on the expression of *Foc* TR4 genes involved in steroid and glycerophospholipids metabolism, we suggest that damage to cell membrane integrity might be a critical reason for the toxicity of DT against *Foc* TR4.

## Conclusion

**FIGURE 8:**
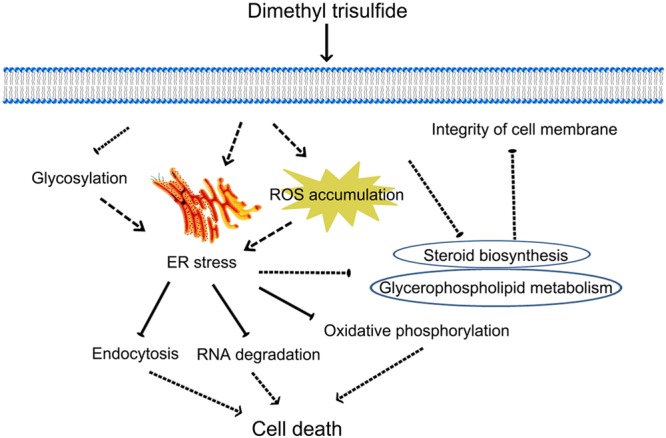
Model illustrating the main molecular mechanism of the response of *Foc* TR4 to DT.

We present a possible mechanism for the toxicity of DT against *Foc* TR4 (**Figure 8**). ER stress of *Foc* TR4, which is related to glycosylation and ROS accumulation, was induced at the early stage of DT exposure; this also caused multiple toxic responses such as RNA degradation, autophagy, and mitochondrial damage. In addition, clear inhibition of the genes involved in steroid biosynthesis and glycerophospholipids metabolism indicated that cell membrane integrity might be disrupted in *Foc* TR4 in response to DT exposure. These data provide a comprehensive high-resolution analysis of the gene expression shifts associated with DT exposure and suggest a new approach for the control of FWB.

## Author Contributions

YH, BC, and CZ conceived the study and participated in its design and coordination. CZ and WZ performed the experiments, collected, analyzed, and deposited the data and prepared the draft. CZ and ZC proofread the final draft and revised the manuscript. All authors have read and approved the manuscript.

## Conflict of Interest Statement

The authors declare that the research was conducted in the absence of any commercial or financial relationships that could be construed as a potential conflict of interest.

## Footnotes

1 https://www.broadinstitute.org/

2 http://primer3.ut.ee/

## Supplementary Material

TABLE S1 Primers used for qRT-PCR.

TABLE S2 Expression profiles of the DEGs related to oxidative phosphorylation, endocytosis, and RNA degradation.

TABLE S3 Significantly DEGs in control and DT-treated groups.

## Abbreviations

BP: Biological process
CC: Cellular component
DEGs: Differentially expressed genes
DT: Dimethyl trisulfide
ER: Endoplasmic reticulum
FDA: Fluorescein diacetate
*Foc*: *Fusarium oxysporum* f. sp. *Cubense*
*Foc* TR4: *Fusarium oxysporum* f. sp. *cubense* tropical race 4
FWB: Fusarium wilt of banana
GO: Gene ontology
KEGG: Kyoto Encyclopedia of Genes and Genomes
MF: Molecular function
PBS: phosphate buffer solution
PDB: potato dextrose broth
qRT-PCR: Quantitative real-time PCR
RNA-seq: RNA-sequencing
ROS: Reactive oxygen species
VCGs: Vegetative compatibility groups
