# Supplementary material for: RNA Sequencing Reveals that Endoplasmic Reticulum Stress and Disruption of Membrane Integrity Underlie Dimethyl Trisulfide Toxicity against Fusarium oxysporum f. sp. cubense Tropical Race 4
Source: Front Microbiol. 2017 Jul 24;8:1365. doi: 10.3389/fmicb.2017.01365 (PMC5522862; doi:10.3389/fmicb.2017.01365)
Supplement: TABLE S1 — Primers used for qRT-PCR. [file Table_1.docx]

**Supplementary table 1: Primers used for qRT-PCR.**

| Primer Name | Sequence (5′-3′) |
| --- | --- |
| act1-1F | CTCCCATCAACCCCAAGTCC |
| act1-1R | AGAAAGTGTAACCGCGCTCA |
| 00414-F | TATGGCGCAACGTTCCCAGG |
| 00414-R | TGGCGGTGTTGGTTGCGAGT |
| 02195-F | GCGAGTACCATTTCCCCGCCTT |
| 02195-R | TACGGTTATGTCGCGCGTAGGC |
| 02810-F | TTCAGCCGCAGTGCCAAAGG |
| 02810-R | AGCCGCTAACACTGGACGCA |
| 05398-F | CGAGGGTATTGGCAAGAACG |
| 05398-R | AGACTCTCCTCGACCTCCTT |
| 06064-F | AGATCGGCTGGGTCACGGTT |
| 06064-R | TGGTGCCACGACGCTGAGAT |
| 07540-F | TGGCCCATGGACCCTAAGCT |
| 07540-R | CCTCACGTCGGAACTTGGCGAT |
| 08157-F | TCTCGCAAACCAGGGCCGAT |
| 08157-R | CCGTAGCAAGGCATGGCATCGA |
| 08856-F | TTCACTGGCTTCGACCGTAT |
| 08856-R | GAAAGTGCCCAAATCCGGTT |
| 10136-F | GCAGCCACGAGAAGAAACCTGG |
| 10136 -R | TGCGCATCGACCATTTCCGC |
| 12358-F | CGCCCAGATGTTCAAGATCG |
| 12358-R | TCCAACAAGCTTCCAATGCC |
| 13609-F | TGGCTACGGAACACCCCAACA |
| 13609-R | AAAGCTGGTTGATGCGGGGC |
| 14808-F | CTTCGAGCAGCTTGACCATC |
| 14808-R | AGCTGGAGATGCAGGTTCTT |
| 15550-F | TCCCGAAGCTGGCGTTGTTG |
| 15550-R | ACGTTTGAGCCGCACCAGAAG |
| 16063-F | ATAGCGATGTGATCACCCGT |
| 16063-R | GAAGCAAACGCGGATCATCT |
| 16340-F | ACGGCGCACCTACTGATGCT |
| 16340-R | AGCCTCCACTGGACATTCTGCC |
